# Broad-range potential of *Asphodelus microcarpus* leaves extract for drug development

**DOI:** 10.1186/s12866-017-1068-5

**Published:** 2017-07-14

**Authors:** Amalia Di Petrillo, Antonella Fais, Francesca Pintus, Celestino Santos-Buelga, Ana M. González-Paramás, Vincenzo Piras, Germano Orrù, Antonello Mameli, Enzo Tramontano, Aldo Frau

**Affiliations:** 1 Department of Life and Environmental Sciences, University of Cagliari, Cittadella Universitaria, SS 554, Bivio per Sestu, I-09042 Cagliari, Monserrato Italy; 2 Grupo de Investigación en Polifenoles, Unidad de Nutrición y Bromatología, Facultad de Farmacia, Universidad de Salamanca, Salamanca, Spain; 3 Department of Surgical Science, University of Cagliari, 09124 Cagliari, Italy

**Keywords:** *Asphodelus microcarpus*, Plant extract, Antibiotics, Biofilm, Antimicrobial activity, Antiviral response, Ebola virus

## Abstract

**Background:**

Many plants have been used in traditional medicine for their antibacterial, antifungal, antiprotozoal, antiviral, antidiarrhoeal, analgesic, antimalarial, antioxidant, anti-inflammatory and anticancer activities.

In order to find novel antimicrobial and antiviral agents, the aim of the present study was the evaluation of the antibacterial and antibiofilm susceptibility of *Asphodelus microcarpus* leaves extract. Moreover, the antiviral activity and the phytochemical composition of the active extract were also determined.

**Methods:**

Antimicrobial and antibiofilm activities of leaves ethanol extract of *A. microcarpus* were evaluated on 13 different microbial strains. We selected three different sets of microorganisms: (i) Gram-positive bacteria, (ii) Gram-negative bacteria and (iii) yeasts. The potential antiviral activity of *A. microcarpus* leaves ethanol extract was evaluated with a luciferase reporter gene assay in which the dsRNA-dependent RIG-I-mediated IFN-β activation was inducted or inhibited by the Ebola virus VP35 protein. HPLC-DAD-MS was used to identify phenolic profile of the active extract.

**Results:**

*A. microcarpus* leaves extract showed a potent inhibitory activity on Gram-positive bacteria while only a reduced inhibition was observed on Gram-negative bacteria. No activity was detected against Yeasts. The extract also showed an interesting antibiofilm motif on various bacterial strains (*E. coli, S. aureus, S. haemolyticus* and *B. clausii*). Moreover, this extract significantly affected the Ebola virus VP35 inhibition of the viral RNA (vRNA) induced IFN response.

**Conclusions:**

The overall results provide supportive data on the use of *A. microcarpus* as antimicrobial agent and a potential source of anti-viral natural products.

Data collected set the bases for further studies for the identification of single active components and the development of new pharmaceuticals.

## Background

The interest for plant extracts and derived compounds is significantly increased over the last years, in the attempt to identify new active substances with therapeutic properties [1, 2]. Plants are rich in a wide variety of secondary metabolites such as tannins, terpenoids, alkaloids, flavonoids, glycosides, which have been found to have in vitro antimicrobial properties [3, 4]. In fact, they could be a good alternative to synthetic chemical antimicrobial agents and antibiotics, because of the serious side effects, antimicrobial resistance and the emergence of previously uncommon infections, which have been increasing due to inappropriate or widespread overuse of antimicrobials. There are several reports in the scientific literature describing the antimicrobial properties of crude extracts prepared from plants [5, 6] and recent studies have been carried out on their anti-biofilm activities against different types of microorganisms [7]. Biofilm is a complex matrix of microorganisms in which cells bind together and attach to biotic or abiotic surface [8, 9]. It has been observed that biofilm formation is associated with many infectious diseases and is a major concern for immunocompromised patients [10] because it provides structural stability and protection to the bacteria, so that the entrapped bacteria become resistant against extreme environmental conditions (i.e. pH, temperature and presence of antibiotics).

Plant extracts show many biological activities [11–14] and they stand as an infinite resource for drug development, novel pharmacophores, and scaffolds for amplification into efficacious drugs for a multitude of disease [1, 2].

A number of small molecules extracted from plants are known for their antiviral effects, while no antiviral drugs coming from plant constituents have been approved so far. For instance, flavonoids and anthraquinones showed inhibitory activity against Influenza A virus, Hepatitis C virus and HIV [15–20]. Defense mechanisms against invading pathogens are accurately controlled by both innate and adaptive immunity. The innate immune system, in particular, is crucial to initiate these anti-pathogen immune activities of the host organism. The type I interferon (IFN) system is a major player in antiviral defense against all kinds of viruses. Virus-infected cells synthesize and secrete IFN that stimulates susceptible cells to express potent antiviral mechanisms that limit further viral growth and spread [21]. However, highly pathogenic viruses such as Ebola virus (EBOV), developed a number of strategies for counteracting innate immune system responses [22, 23]. In particular, the EBOV VP35 protein is essential for viral inhibition of IFN production and, hence, it has been shown to be an effective viral target [23, 24]. Within this context, one approach to subvert this powerful immune response inhibition is to identify small molecules that potentiate or activate the IFN signaling pathway, increasing IFN production in response to viral infections to a level able to overturn this inhibition.

Mediterranean area offers a great variety of endemic plants in Europe and, due to its geographical isolation, the island of Sardinia provides an even greater plant biodiversity, with significant variations in genetic and molecular characteristics as compared to plants grown in other regions.

The primary benefit of plant-derived medicines is due to their availability, fewer side effects and reduced toxicity. Ethnopharmacological studies have shown that a number of them were used until less than 100 years ago in folk medicine [25] and some of them were reported to have therapeutic effects on inflammations and immune system reinforcements [26].

*Asphodelus microcarpus* Salzm.et Vivi (Asphodelaceae) is widely distributed over the coastal Mediterranean region and was traditionally used as an antimicrobial agent [27]. In ethnobotanical literature, its use for otitis and toothache in Algeria [28] and for lung diseases in Sardinia [29] has been also reported. Several studies were performed in order to verify its antimicrobial activity [30–32]. Recently, antimicrobial activity of areal part of *A. microcarpus* was evaluated on *Propionibacterium acnes*, implicated in the pathogenesis of acne vulgaris [33], and on methicillin resistant *Staphylococcus aureus* MRSA [34]. The methanolic extract of *A. microcarpus* leaves demonstrated a higher anitimicrobial activity against *Staphylococcus aureus* and *Candida albicans* [35].

*A. microcarpus* is traditionally used for the toothache and this prompted us to investigate the anti-biofilm activity and the potential antimicrobial activities of *A. microcarpus* leaves extract against oral and environmental bacteria frequently reported in dental unit water line (*Streptococcus spp., Staphylococcus spp., Escherichia coli*) [36].

Moreover, based on previous experiments described in different publications [37, 38], potential antiviral activity of *A. microcarpus* extract have been carried out.

## Methods

### Plant collection

*Asphodelus microcarpus* subsp. *microcarpus* Salzm. et Viv. (syn. *Asphodelus ramosus* L. subsp. *ramosus*) leaves were collected in southern Sardinia (Quartu Sant’Elena, Cagliari, Italy). The GPS coordinates were 39° 22′41.5″ N and 09° 19′62.3″ E. The plant was identified by Dr. Cecilia Loi, Department of Life and Environmental Sciences, Section of Botany, University of Cagliari, Italy. A voucher specimen (1405/16 Herbarium CAG) has been deposited in the Museum Herbarium CAG (Life and Environmental Sciences Department).

Plant materials were washed with deionized water, frozen at −80 °C and then lyophilized. The dried plant was stored at −80 °C until required.

### Extraction procedure

The lyophilized plant materials (10 g) were extracted in 50 mL of ethanol for 24 h at room temperature under continuous stirring. After filtration, ethanol extract was diluted 10-fold with water and then lyophilized (*Asphodelus* Extract, AE). Dried powder was dissolved in 10% DMSO for antimicrobial activity tests. For HPLC–DAD–ESI/MS analyses, dried extract was dissolved in 1 mL of 0.1% formic acid: acetonitrile (70:30, *v*/v) and filtered through a 0.22 μm disposable LC filter disk for HPLC analysis.

### Microbial strains

To evaluate the antimicrobial profile of AE, 13 different microbial strains have been used. We selected three different sets of microorganisms: (i) Gram-positive bacteria, *Staphylococcus aureus* ATCC 6538 (American Type Culture Collection), *Staphylococcus haemoliticus* clinical isolate NC1, *Streptococcus uberis* human clinical isolate NC 20, *Streptococcus faecalis* ATCC 29212, *Streptococcus mutans* CIP103220 (Collection Institut Pasteur), *Streptococcus salivarius*, strain k12 (from a commercial product, Bactoblis®), *Streptococcus pyogenes* human clinical isolate NC4, *Streptococcus intermedius* DSMZ 20573 (German Collection of Microorganism and cell culture), *Bacillus clausii*, (isolated from a commercial product, Enterogermina®); (ii) Gram-negative bacteria, *Escherichia coli* ATCC 7075; (iii) Yeasts, *Candida albicans*, *Candida kruseii*, *Candida glabrata*, human oral clinical isolates, designed from BF1 to BF3 respectively. *E. coli*, *B. clausii*, *S. haemolyticus* and *S. aureus* were plated in Muller Hinton agar; Shaedler agar for Streptococci and Sabouraud dextrose agar for fungi. Prior to use, all the strains were stored in a tube containing the proper medium broth with 20% of glycerol at −80 °C. These microbial strains were used for an in vitro susceptibility test: (a) the agar diffusion method, (b) Minimal Inhibitory Concentration (MIC), (c) Minimum Bactericidal Concentration (MBC), which were determined in accordance with the National Committee for Clinical Laboratory Standards, NCCLS. In addition, the Minimal Biofilm Inhibitory Concentration (MBIC) was used to evaluate the AE antibiofilm activity [39].

### Antimicrobial susceptibility testing

The Agar diffusion method was performed by using the Kirby-Bauer (KB) procedure and used as preliminary antimicrobial test to reveal the entire antimicrobial susceptibility profile for the examined AE. 1·10^7^ cells/mL were inoculated onto the surface of an agar plate containing one of the subsequent bacterial growth agar mediums (Microbiol, Uta, Italy): (i) Muller-Hinton agar for aerobic bacteria, (ii) Shaedler agar for Streptococcus spp., (iii) Fungi on Sabouraud agar. This antimicrobial activity test was performed following the NCCLS protocol by using a paper filter disc (Ø = 6 mm) impregnated with AE work solution (1000 μg/mL). MIC and MBC were performed only in susceptible microbial strains with KB test and they were performed according to the Micro-broth dilution method [40, 41] by using a ½ serial dilution, from 500 to 3.9 μg/mL of the AE; the positive controls were performed with a Chlorhexidine digluconate solution (CHX), Sigma-Aldrich, ranged a concentration from 500 to 0.48 μg/mL (Table 1). The cultures were incubated in air at 37 °C for 24 h for the aerobic strains and in 5% CO_2_ at 37 °C for the Streptococcal species. For the biofilm evaluation, we used the protocol described by Montana University’s Center for Biofilm Engineering [42]. A microplate containing serial concentrations of the compound, inoculated with the bacterial strains as previously described for MIC and MBC evaluation, was incubated at 37 °C for 6 days to permit the biofilm formation. The plate samples were subsequently washed three times with Phosphate-buffered saline GIBCO®PBS (ThermoFisher) to eliminate planktonic cells; thus, the biofilm was stained with 100 μL of 0.1% *w*/*v* of crystal violet solution (Microbial, Uta, Italy) for 10 min at 25 °C. After three washes with PBS solution, 200 μL of 30% *v*/v acetic acid was added in every well to solubilize the dye from the bacterial biomass. The biofilm amount was measured with a plate reader spectrophotometer (SLT-Spectra II, SLT Instruments, Germany) at 450 nm. The experiment was performed in triplicate and the MBIC represented the lowest concentration showing a 450 nm absorbance comparable with negative control, > 95% (sample without bacteria).

**Table 1:** Antibacterial profile of *A. microcarpus* leaves extract on a set of different microorganisms

Strains	Kirby-Bauer Ø mm	^a^MIC (μg/mL)	MBC (μg/mL)
Gram-positive bacteria			
*Bacillus clausii*	4 ± 1	250	>500
*Staphylococcus aureus*	10 ± 2	250	>500
*Streptococcus salivarius*	-^b^	-^b^	-^b^
*Streptococcus mutans*	-^b^	-^b^	-^b^
*Staphylococcus haemolyticus*	6 ± 1	250	>500
*Streptococcus faecalis*	-^b^	-^b^	-^b^
*Streptococcus intermedius*	-^b^	-^b^	-^b^
*Streptococcus pyogenes*	-^b^	-^b^	-^b^
*Streptococcus uberis*	-^b^	-^b^	-^b^
Gram-negative bacteria			
*Escherichia coli*	4 ± 1	500	>500

^a^MIC values with CHX were ranged for all strains from 7.81 (*S. aureus*) to 3.9 (*E. coli*) μg/mL

^b^(−) strain that resulted no sensitive with preliminary KB antimicrobial test Ø = 0 mm, conc. 1000 μg/mL

### Cell line and viral infection

A549 cells were propagated in DMEM (Gibco™) supplemented with 10% Fetal Bovine Serum (E.U.-approved, South America Origin, Gibco™) and 1% penicillin/streptomycin (Euroclone®). Cells were incubated at 37 °C in humidified atmosphere of 5% CO_2_ and 95% air. For production of vRNA, A549 cells were infected with Influenza virus A/Puerto-Rico/8/34 (H_1_N_1_) strain (IAV/PR/8/34) with a multiplicity of infection of 5. Five hours after infection, total RNA was isolated using the RNeasy Kit (Qiagen).

### Cytotoxicity assay

The effect of AE on A549 cells proliferation was determined in 96-well plates (Spectra Plate, PerkinElmer). Cells were seeded at initial density of 10^5^ cells/mL and cultured in DMEM (Gibco™) supplemented with 10% Fetal Bovine Serum (E.U.-approved, South America Origin, Gibco™) and 1% penicillin/streptomycin (Euroclone®), in the presence or absence of serial dilutions of extract (ranging from 100 μg/mL to 0.03 μg/mL). Camptothecin was used as positive control Table 2. Plates were incubated for 72 h at 37 °C in humidified atmosphere of 5% CO_2_ and 95% air. Cell viability was determined adding PrestoBlue™ Cell Viability Reagent (Invitrogen). Following 1 h incubation at 37 °C, relative fluorescence was read with a Victor3 (Perkin Elmer). The percentages of cell viability were calculated on the amount of living cells in extract treated cells relative to untreated control cells (defined as 100% viability). Cytotoxicity graph was then generated by plotting percentage of cell viability versus concentration of extract. The concentration required to reduce cell growth by 50% (CC_50_) was calculated using regression analysis of cytotoxicity curves.

**Table 2:** Cell growth inhibition of A549 cell line by plant extract. Data shown are the mean ± SD of three independent experiments performed in duplicate samples

Extract/compound	^a^CC_50_ (μg/mL)
AE	>100
Camptothecin	0.54 ± 0.15

^*a*^ *Extract/compound concentration required to reduce cell growth by 50%*

### Luciferase reporter gene assay for measuring IFN-β induction

The luciferase reporter gene assay was performed as previously described [37]. Briefly, A549 cells (5 × 10^4^ per well) were transfected with T-Pro P-Fect Transfection Reagent (T-Pro Biotechnology, Twin Helix) and with the construct pGL IFN-β luc. Twenty-four h after transfection, cells were additionally transfected with Influenza virus A/PR/8/34 (H_1_N_1_) and incubated for further 6 h at 37 °C with 5% CO_2_ in presence or absence of the extract. Then, cells were harvested with a Luciferase Harvesting Buffer (50 mM Na-MES pH 7.8, 50 mM TrisHCl pH 7.8, 1 mM dithiothreitol, 0.2% Triton X-100). The crude cell lysates were clarified by centrifugation and 50 μL of cleared lysates were added to 50 μL of Luciferase Assay Buffer (125 mM Na-MES pH 7.8, 125 mM Tris–HCl pH 7.8, 25 mM magnesium acetate, 2.5 mg/mL ATP) in a white 96-well plate (OptiPlate, PerkinElmer). Immediately after addition of 50 μL of 1 mM D-luciferin into each well, the luminescent signal was measured in Victor3 luminometer (Perkin Elmer). The relative light units measured were normalized as the fold activity of the unstimulated control. Each assay was carried out in triplicate.

### EBOV VP35 luciferase reporter gene inhibition assay

The above described luciferase reporter gene assay was also performed for evaluating the IFN-β induction inhibition mediated by EBOV VP35 protein. When the pcDNA3-ZEBOV-VP35 was used as control, it was co-transfected with the pGL IFN-β luciferase plasmid. Inhibition of luciferase expression was indicated as percentage of induced control. Each assay was carried out in triplicate.

### HPLC-DAD-ESI/MS analysis

The AE was analyzed as previously reported [43]. Briefly, the solvents were: (A) 0.1% formic acid, and (B) acetonitrile. The elution gradient established was isocratic 15% B for 5 min, 15–20% B over 5 min, 20–35% B over 10 min, 35–50% B over 10 min, 50–60% B over 2 min, isocratic 60% B for 5 min and re-equilibration the column, using a flow rate of 0.5 mL/min. Double online detection was carried out in the DAD using 280 nm and 370 nm as preferred wavelengths and in the MS operated in the negative ion mode. The phenolic compounds present in the samples were identified according to their UV and mass spectra and by comparison with commercial standards, when available.

### Data analysis

Data are expressed as mean ± SD from three independent experiments. The analysis average of the treatment was determined using *t-*student (Prism 6 program), and the data were compared using the *p* values: *p* < 0.05 was considered statistically significant.

## Results

### Antimicrobial susceptibility testing and citotoxicity of *A. microcarpus* extract

**Fig. 1:**
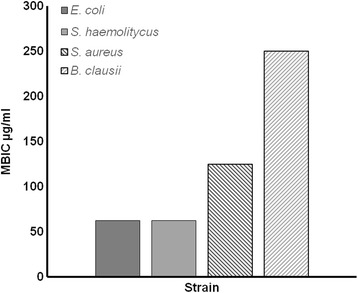
Minimal biofilm inhibitory concentration (MBIC) on four bacterial strains resulted biofilm sensitive at *A. microcarpus* extract. MBIC represent the lowest concentration showing a 450 nm absorbance >95% comparable with negative control, sample without bacteria (ABS, SD ±15%). The CHX showed a MBIC values ranged from 3.9 to 0.97 μg/mL

When tested on 13 different microorganisms, AE showed an inhibitory effect on Gram-positive bacteria while lower inhibition was observed on the Gram-negative bacteria *E. coli* (Table 1). When tested for antibiofilm activity, AE showed an interesting effect on various bacterial strains (*E. coli, S. aureus, S. haemolyticus and B. clausii*). The MBIC was shown to be, except for *B. clausii*, 2 or 4 fold lower than the respective MIC value, Fig. 1. This result suggests that AE is able to counteract the biofilm formation by the probable presence of anti-attachment or quorum quenching substances [44]. No activity was detected against yeasts (data not shown).

To evaluate the AE effects on the EBOV VP35 inhibition of the IFN activation, we first screened AE for eukaryotic cellular toxicity in order to determine the appropriate concentrations for the following luciferase reporter IFN-β gene assays. Total AE showed no cytotoxic effects on A549 cells with a CC_50_ > 100 μg/mL. Camptothecin was used as positive control. Considering these results, the initial concentration of 30 μg/mL was chosen to investigate the effects of a possible increase of the dsRNA RIG-I-mediated IFN-β induction.

### Potential antiviral activity of *A. microcarpus*

**Fig. 2:**
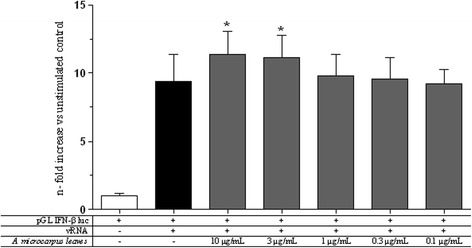
*A. microcarpus* leaves extract effects on IFN-β induction. The AE was tested in a RIG-I-mediated IFN-β induction system with vRNA stimulation. Results are shown in n-fold compared to unstimulated control (* *p* value <0.05)

EBOV VP35 is a validated drug target involved in several crucial processes for a successful viral replication and propagation. In order to study the AE capacity to potentiate the dsRNA-dependent RIG-I-mediated IFN-β induction, the luciferase reporter gene assay and the VP35 inhibition assay previously described were performed [37]. Results showed that AE was capable to significantly potentiate (*p* value <0.05) dsRNA-dependent RIG-I-mediated the IFN-β production at the concentrations of 10 μg/mL and 3 μg/mL (Fig. 2). Noteworthy, AE did not stimulate the innate antiviral response when tested in the absence of a vRNA stimulus (data not shown).

Given the ability of increasing IFN production following dsRNA stimulation shown by AE in the IFN-β induction assay, we then investigated whether such increase of IFN-induction was adequate to subvert the blockade of the signaling cascade mediated by EBOV VP35. For this reason, the same AE concentrations were tested in dsRNA-dependent RIG-I-mediated IFN-β induction system in the presence of EBOV VP35.

**Fig. 3:**
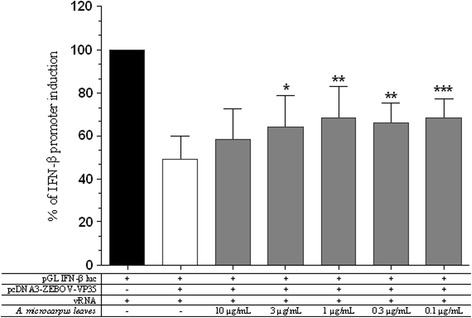
*A. microcarpus* leaves extract effects on IFN-β induction inhibition of EBOV VP35. The extract was tested in RIG-I-mediated IFN-β induction system in presence of EBOV VP35 inhibition. Results are shown as the percentage of IFN-β promoter induction (* *p* value <0.05, ** *p* value <0.01, *** *p* value <0.001)

Results showed that AE significantly reverted the EBOV VP35 inhibition of the vRNA induced IFN response at concentrations of 3–0.1 μg/mL (3 μg/mL *p* < 0.05, 1 and 0.3 μg/mL *p* < 0.05, 0.1 μg/mL *p* < 0.001) (Fig. 3).

### Characterization of phenolic compounds in *A. microcarpus* leaves extract

**Fig. 4:**
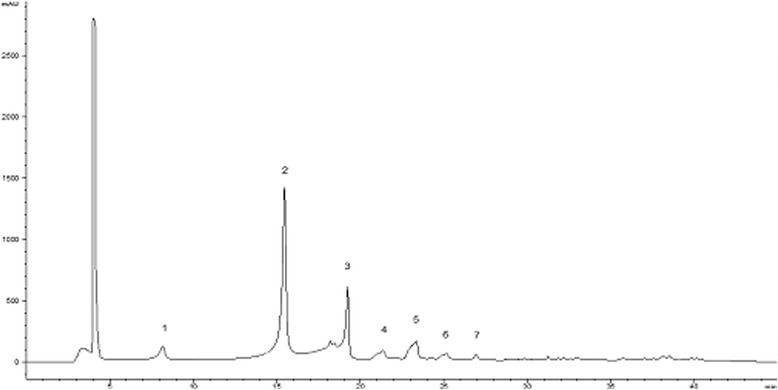
HPLC chromatogram of *A. microcarpus* leaves recorded at 330 nm for phenolic compounds. Peak identification is given in Table 3

The HPLC phenolic profile of *A. microcarpus* leaves extract, recorded at wavelength, *λ* = 330 nm, is shown in Fig. 4. Data on retention times, (*t*_r_), wavelengths of maximum absorbance (*λ*_max_), pseudo-molecular ions ([M-H]^−^), diagnostic fragments and tentative identification for each peak of the phenolic compounds detected are listed in Table 3. Results showed a phenolic profile similar to the previously described in *A. microcarpus* flowers [43] but, in this case, the major peak 2 occurs with luteolin-6-C-glucoside. Moreover, cichoric acid and cumaril exosa malic acid were not present in flowers extract.

**Table 3:** Identification of polyphenolic compounds in *A. microcarpus* leaves extract by HPLC-DAD- ESI/MS analysis

Peak	Rt (min)	λmax (nm)	Molecular ion [M-H]^−^ (*m/z*)	MS^2^(*m/z*), (%)	Tentative identification
1	8.1	326	353	191(100) 354(50)	5-*O*-caffeoylquinic acid
2	15.4	350	447	429(30) 357(94) 327(95) 447(100)	Luteolin −6-*C*- glucoside
3	19.3	350	489	429(11) 357(38) 327(91) 489(100)	Luteolin −6-*C*- acetilglucoside
4	21.2	336	473	311(100) 341(27) 283(17) 413(12) 429(3) 473(93)	Cichoric acid
5	23.2	332	623	623(100) 447(58) 429(38) 309(26) 417(24) 327(18)	Luteolin-*C*-glucoside
6	25.1	332	441	441(100) 213(48) 153(9) 195(8) 399(4)	Cumaril exosa malic acid
7	27	336	285	285(100)	Luteolin

## Discussion

*Asphodelus microcarpus* leaves showed the ability to inhibit some Gram-positive and, with lower potency, Gram-negative bacteria but displayed no activities against *Streptococci spp*. and yeasts.

If we compare two Gram positive bacteria groups, our data demonstrate that AE extract is more active with aerobic strains (Staphylococcus) than with facultative anaerobic strains (Streptococcus). Following previous reports, these differences in sensitivity could be due to different cytoplasmic cell membrane composition. In fact, flavonoids are able to interfere with different cell membrane compounds in lipid bilayers and they may control the arrangement of membrane proteins with the formation of functional complexes responsible for cell signal transduction and the regulation of the metabolism [45]. In this context by using luteolin, different authors reported decreases in ATPase activity and in the production/secretion of α-toxin in *Staphylococcus aureus* [46, 47].

The antimicrobial activity of ethanolic extract from *A. microcarpus* tubers has been previously published [32]. Moreover, a previous study reported the antimicrobial activities of methanolic extract of *A. microcarpus* leaves [35]. Our results indicated that ethanolic extract of *A. microcarpus* exhibited a different antimicrobial activity against *E. coli*, *S. aureus* and *Candida albicans* if compared with previous report [35]. It is considered that these differences depend on solvent material and extraction process used.

In the present study, MBIC test was carried out to evaluate the possible effects of AE on biofilm-mediated diseases: results showed an antibiofilm effect against some pathogens (*E. coli, S. aureus, S. haemolyticus and B. clausii*). Further studies are required to explain if the AE acts against biofilm formation through the quorum-sensing pathway or it acts as an anti-attachment agent.

The discovery of small molecules able to reinforce the innate antiviral response when it is under viral attack is a novel and powerful approach against the inhibitory strategies carried out by highly pathogenic viruses such as EBOV through its VP35 protein. For this purpose, we tested *A. microcarpus* leaves extract to verify if it was capable to modulate the dsRNA-dependent RIG-I-mediated IFN-β induction. It is noteworthy that the AE did not stimulate the innate antiviral response when tested in the absence of a vRNA stimulus and showed the ability to potentiate the viral-induced IFN-β production. Significantly, AE showed to be able to subvert VP35 effects at different effective concentrations with an almost similar efficacy between 3 and 0.1 μg/mL and a lower effect at the higher dose of 10 μg/mL. It is worth of note that the window of AE efficacy in subverting VP35 inhibition of the IFN production is not completely overlapping with the AE dose–response IFN production shown in Fig. 2. Even if the reason of this is not clear at the moment, it is possible to speculate that the AE interaction with the IFN production pathways could be different in the presence of the viral protein. Smaller AE concentrations could be effective in reverting the VP35 inhibition but the IFN production system used could not be sensitive enough in the absence of VP35. The VP35 reverting activity, along with the fact that *A. microcarpus* leaves extract acts only under antiviral response induction, makes this plant extract very attractive for the development of an antiviral drug. Indeed, further studies will be required to: i) identify the constituent of the extract responsible for the antiviral activity and ii) determine the extract’s mode of action that, presumably, interacts with one or more components of the RIG-I-mediated IFN signaling pathway increasing IFN production. In fact, as shown by HPLC-DAD- ESI/MS analysis (Fig. 4), *A. microcarpus* extract is a complex matrix of several molecules, that could possibly act together inducing IFN production interacting with different cellular targets, as proposed for other synthetic compounds [48]. It is important to note that phenolic characterization of *Asphodelus microcarpus* leaves showed high presence of luteolin derivatives. These flavones were extracted from many plants and several studies showed their anti-inflammatory, antibacterial, antioxidant and anti-HIV activity and may be involved in AE biological activity [49–51].

In addition, luteolin has been shown to inhibit the kinase activity of TBK [52] a component of the RIG-I cascade. Hence, it is possible that either luteolin and/or luteolin-*C*-glucoside may modulate dsRNA-dependent RIG-I-mediated IFN-β production. Further studies will be needed to investigate these possibilities. Overall, additional studies will be also needed to characterize extract fractions in order to identify the individual molecules with biological activity.

## Conclusion

Nowadays, natural products appear to be an interesting solution against the emergency of the antibacterial resistance and immune-regulators. In this study, *A. microcarpus* extract has shown a useful antibiofilm effect that could have a crucial role against biofilm-mediated diseases. However, other studies will be needed to clarify AE active component as well as the AE mechanism in the biofilm formation and to verify if others bacteria are sensitive to AE.

Finally, given that EBOV VP35 is a paradigm protein for potent viral IFN-suppression, the present study represents an interesting starting point for further studies that should focus on: i) the study of the mechanism of action of *A. microcarpus* extract and its constituents in the dsRNA-dependent RIG-I-mediated IFN signaling pathway, in order to potentiate the innate immune response; ii) the design and synthesis of molecules as selective inhibitors/revertant of EBOV VP35 inhibition of IFN production. In the need for new available antiviral therapeutics of natural origin, *A. microc*arpus appears to be a promising starting point for the development of molecules with broad antiviral activity.

## Abbreviations

AE: *Asphodelus* extract
CHX: Chlorhexidine digluconate solution
EBOV: Ebola virus
HCV: Hepatitis C virus
IFN: I interferon
KB: Kirby-Bauer
MBC: Minimum bactericidal concentration
MBIC: Minimal biofilm inhibitory concentration
MIC: Minimal inhibitory concentration
vRNA: viral RNA
